# Periodical Microstructures Based on Novel Piezoelectric Material for Biomedical Applications

**DOI:** 10.3390/s151229876

**Published:** 2015-12-15

**Authors:** Giedrius Janusas, Sigita Ponelyte, Alfredas Brunius, Asta Guobiene, Igoris Prosycevas, Andrius Vilkauskas, Arvydas Palevicius

**Affiliations:** 1 Faculty of Mechanical Engineering and Design, Kaunas University of Technology; Studentu str. 56, Kaunas LT-51424, Lithuania; giedrius.janusas@ktu.lt (G.J.); alfredas.brunius@ktu.edu (A.B.); igoris.prosycevas@ktu.lt (I.P.); andrius.vilkauskas@ktu.lt (A.V.); arvydas.palevicius@ktu.lt (A.P.); 2 Institute of Materials Science, Kaunas University of Technology, Barsausko str. 59, Kaunas LT-51423, Lithuania; asta.guobiene@ktu.lt

**Keywords:** PZT, piezoelectric effect, periodical microstructure, SPR

## Abstract

A novel cantilever type piezoelectric sensing element was developed. Cost-effective and simple fabrication design allows the use of this element for various applications in the areas of biomedicine, pharmacy, environmental analysis and biosensing. This paper proposes a novel piezoelectric composite material whose basic element is PZT and a sensing platform where this material was integrated. Results showed that a designed novel cantilever-type element is able to generate a voltage of up to 80 µV at 50 Hz frequency. To use this element for sensing purposes, a four micron periodical microstructure was imprinted. Silver nanoparticles were precipitated on the grating to increase the sensitivity of the designed element, *i.e.*, Surface Plasmon Resonance (SPR) effect appears in the element. To tackle some issues (a lack of sensitivity, signal delays) the element must have certain electronic and optical properties. One possible solution, proposed in this paper, is a combination of piezoelectricity and SPR in a single element.

## 1. Introduction

In the past few decades biomedical applications have required fast, reliable, miniature and low-cost methods and tools for recognition of diverse biomolecules in various fluids. One of the recent new applications in this area is related to biosensing elements based on cantilever type sensing platforms. These platforms are able to convert biological responses into electrical signals [1,2]. Great potential in biosensing application areas has been found not only for cantilever sensors [3,4,5], but also for film bulk acoustic resonators (FBAR) [6]. 

This paper discusses a cantilever-type sensing element design made of a novel piezoelectric material exhibiting high resonance frequencies, leading to a faster response time and much higher sensitivity compared to cantilevers made of silicon [7], silicon oxide [8], *etc*. The advantage of the design proposed in this paper is a periodical microstructure imprinted on top of the piezoelectric layer with metal nanoparticles precipitated on the grating ridges. Because of the incorporation of noble (in this case silver) nanoparticles, the Surface Plasmon Resonance (SPR) effect appears. SPR is a powerful tool for investigating biomolecular interactions with label-free real-time analytical technologies. This effect greatly influences the efficiency, the structure and operation of the sensor, *i.e.*, much greater control of optical properties, sensitivity and selectivity may be achieved. Moreover, to achieve the maximum optical effect, an operating wavelength of the sensing element may be tuned to a spectral region where the SPR peak is sharpest. 

Many various materials have piezoelectricity properties, but only few of them are most promising in MEMS and NEMS technologies, *i.e.*, zinc oxide (ZnO) films [9,10], polyvinylidene fluoride (PVDF) films [11,12] and lead zirconate titanate (PZT) [13,14], the so-called polycrystalline ceramics. These three main materials have high piezoelectric coefficients, very good flexibility and strong electromechanical coupling. Moreover, it is known that nano-sized ceramics are very different compared to bulk ceramics in their mechanical behavior. This research paper concentrated on designing a novel piezoelectric material, working at low frequencies and able to harvest energy or to cause deformations. This novel material may be integrated in sensing or actuating elements, depending on the purpose of the microsystem. Economical and easy fabrication allows its use in current technologies. In this research paper, PZT (exhibiting high piezoelectric coefficient and permittivity, large dielectric constants and good conversion efficiency) was chosen as a basic material for creating a novel piezoelectric sensing platform. A mixture of 20% polyvinylbutyral together with PZT powder was synthesized. Since strong binding properties are essential, polyvinylbutyral works here as a binding element with PZT ensuring good adhesion and flexibility. Three concentrations (40%, 60% and 80%) of PZT composite materials were produced for in depth investigation. Each PZT material was coated as a thin film and sandwiched between two copper electrode layers. Results showed that in the *d_31_* mode configuration, it harvested energy, *i.e.*, at 50 Hz frequency it generated up to 80 μV. Further, a 4 μm periodical microstructure was imprinted on thin 80% PZT piezoelectric film and silver (Ag) nanoparticles deposited on it. A property of piezoelectricity allows tuning a diffraction grating and results in variation of diffraction efficiencies. This allows one to achieve a desirable spectral region in which the designed cantilever–type sensing platform would display high-efficiency. To perform certain specialized sensing functions it must reliably store and convert different forms of energy, transduce signals, and respond to repeated exposure to external biological and chemical environments. The designed cantilever-type sensing platform can alter its mechanical stress within the oscillator and its total mass when a target analyte is in contact with its surface. Here, a signal transduction is achieved by employing a diffraction grating to measure the mechanical bending or the frequency spectra resulting from additional loading by the absorbed mass. Since both the resonant frequency shift and deflection are highly dependent on the position of the absorbed material (analyte) it is difficult to determine the exact amount of an additional mass. A diffraction grating and incorporation of Ag nanoparticles on its surface allow adequate control of chemical surface functionality for the detection of analytes of interest, *i.e.*, defined molecules can be absorbed from analyte onto the Ag anchors, creating a strong interaction between the functional group and the silver nanoparticles. The vibrating cantilever-based platform offers quantitative assessment of the specific mass when resonant frequency shifts are experimentally monitored. Advantages of the designed novel sensing platform include easy fabrication; inexpensive materials and equipment required; ability to make thin (from 400 nm to 1.4 µm) films; wide application areas (from sensitive diagnostic devices in medicine, pharmacy, to microsystem devices in wireless sensors and portable electronics). An interesting fact is that a few different components―resonance, piezoelectricity, diffraction efficiency and silver nanoparticles—are all combined in a single element. 

## 2. Experimental Section 

The novel piezoelectric sensing platform was created and evaluated in the Institute of Materials Science of Kaunas University of Technology and in the Institute of Mechatronics of Kaunas University of Technology.

### 2.1. Material, Synthesis and Formation

The novel piezoelectric material was designed using PZT powder and a 20% solution of polyvinyl butyral in benzyl alcohol mixed under defined conditions. Three material types using different PZT concentrations (40%, 60% and 80%) were produced. Here, a screen printing technique was used to cover the base of the elements with PZT paste on a copper foil. Using a 325 mesh of stainless steel PZT paste was printed and dried at 100 °C for 30 min. This procedure allows one to apply a smooth and equal thickness of the formed thin films (in this case, the thickness of all three elements was ~1.2 μm). Further, a 4 μm periodical microstructure was formed on the top of piezoelectric thin film by a hot-embossing technique. Ag nanoparticles were formed from a solution of 0.05 M AgNO_3_ in deionized water and dip-coated onto the periodical microstructure. The purpose of using different PZT concentrations is the creation of piezoelectric materials with good piezoelectric characteristics and sufficient elasticity for imprinting a well-defined grating. 

### 2.2. Cantilever Type Piezoelectric Element Structure 

**Figure 1:**
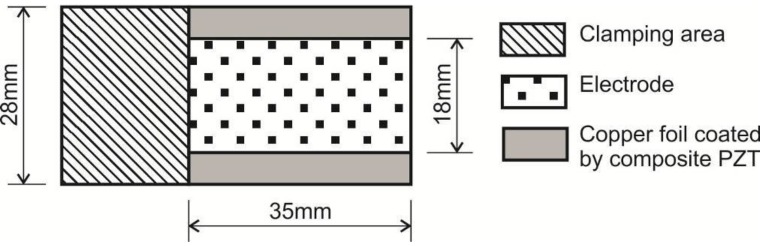
Principal scheme of the piezoelectric element.

A novel cantilever-type sensing element was made. For evaluation of its piezoelectric properties, three samples with different PZT (40%, 60% and 80%) concentrations were designed (Figure 1).

A cantilever-based sensing platform allows precise evaluation of piezoelectric properties. The elements were investigated for both direct and indirect piezoelectric effects. 

### 2.3. Characterization Methods

#### 2.3.1. Surface Morphology and Chemical Composition Measurements

The structure and chemical composition of the designed material was investigated using a Scanning Electron Microscope (SEM, Quanta 200 FEG, Hillsboro, OR, USA), integrated with an Energy Dispersive X-Ray Spectrometer (EDS) X-Flash 4030 detector from Bruker (Berlin, Germany). Samples were examined under a controlled pressure water steam atmosphere. A 133 eV (at Mn K) energy resolution at 100.000 cps is achieved with a 30 mm^2^ area solid state drift detector, cooled with a Peltier element. The X-ray spectroscopy method allows analyzing energy distributions. The energy differences are measured between the various quantum states of a system together with the probabilities that the system jumps between these states. 

Investigations of surface morphology were performed with an Atomic Force Microscope (NT-206 (Micro-test Machines Co., Gomel, Belarus) in contact mode. This is a surface analytical technique employed to generate very high-resolution topographic views of a surface down to molecular/atomic resolution, with the only requirement being that the sample be deposited on a flat surface. This method provides spatial resolutions of 1–20 nm. AFM is able to analyze electrical, magnetic, mechanical and chemical properties in nanoscale dimension, using specialized modes. Main morphology parameters: *Z_mean_*-average height, *R_a_*-arithmetic average surface roughness, *R_q_*-root mean square surface roughness.

#### 2.3.2. Harmonic Excitation Measurements

**Figure 2:**
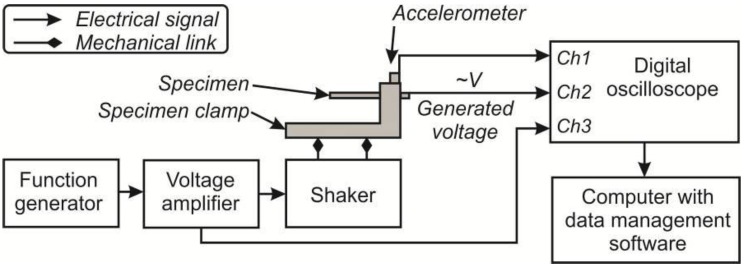
An experimental setup scheme used to measure harmonic excitations of piezoelectric elements.

Harmonic excitation measurements were performed using a scheme depicted in Figure 2. It consists of a piezoelectric element, excitation measurement and data acquisition systems. An electromagnetic shaker excites the element fixed in a custom-made clamp. A harmonic excitation signal is transmitted to the electromagnetic shaker and controlled by a AGILENT 33220 A (Agilent, Santa Clara, CA, USA) function generator and a HQ Power VPA2100MN voltage amplifier (HQ Power, Gavere, Belgium). For acceleration measurements, at the top of the clamp a single–axis KD-35 miniature accelerometer (Metra, Radebeul, Germany) with sensitivity of 50 mV/g ±2% and working frequency from 5 Hz to 5 kHz is attached. Signals from the voltage amplifier, accelerometer and an element are collected with the data acquisition system, consisting of a 4-channel PICO 3424 USB oscilloscope (analog-to-digital converter; Pico, St Neots, UK), and forwarded to a computer. Obtained data is then analyzed with the Pico-Scope 6 software.

#### 2.3.3. Vibration Analysis

**Figure 3:**
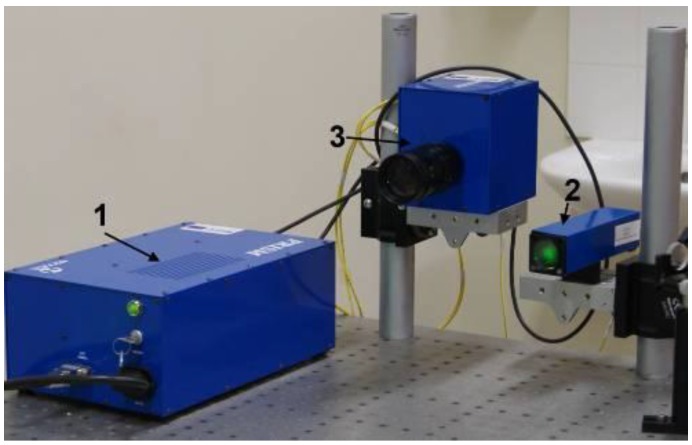
A PRISM measurement system consisting of: (**1**) A control block; (**2**) An object illumination head; (**3**) A video camera.

A two beam speckle pattern interferometer, or so-called PRISM system (measurement sensitivity <20 nm, measurement range >100 μm), was used to evaluate the electrical excitation response of the investigated piezoelectric elements. This method allows measuring vibration and deformation with minimal sample preparation and with no contact with the sample surface. PRISM is a rather high-speed holographic technique, equipped with a computer system and integrated software (Figure 3).

**Figure 4:**
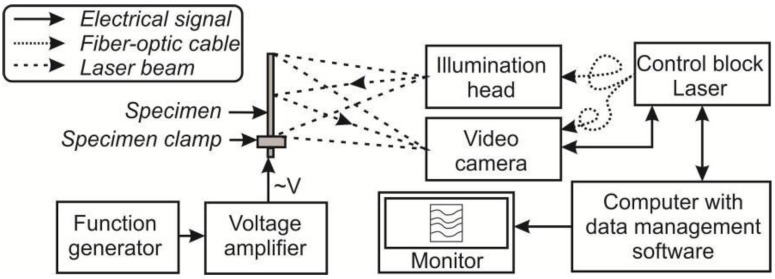
The PRISM experimental set-up.

The laser beam directed to the object is an object beam; the beam directed to the camera is a reference beam (Figure 4). The camera lens collects the scattered laser light from the object and images the object onto the sensors of the CCD camera. The reference beam falls directly to the camera and overlaps the image of the object. The fringes displayed on the monitor appear because of the shape changes occurring between a reference and a stressed state of the object. The obtained data allows evaluating the electrical excitations of the investigated element. 

#### 2.3.4. Measurements of Diffraction Efficiency

For evaluation of the diffraction efficiency in all peaks for different incident angles, a red He-Ne laser diffractometer (wavelength 632.8 nm incident to grating) was used. It consists of a He-Ne laser source, a set of mirrors to direct a beam, a photodiode and a tester, to record the measurement data. A photodiode records the intensity and angle of diffracted light from the grating in all maxima (0, ±1, ±2, and *etc.*), which is dependent on a grating period. 

## 3. Results and Discussion

### 3.1. Surface Morphology of Novel Cantilever Type Piezoelectric Elements

**Figure 5:**
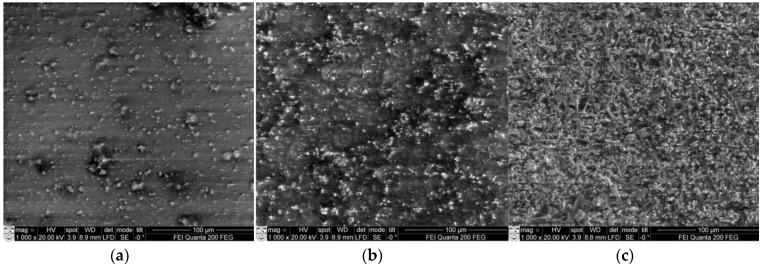
SEM view of piezoelectric elements with PZT concentration of (**a**) 40%; (**b**) 60% and (**c**) 80%.

The surface morphology and chemical properties of three different elements with PZT concentrations of 40%, 60% and 80%, were investigated. The surface morphology of the piezoelectric elements was investigated using Scanning Electron Microscopy (Figure 5). 

**Figure 6:**
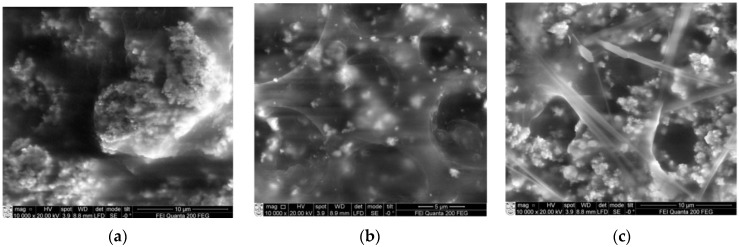
Images of SEM of piezoelectric elements when PZT: (**a**) 40%; (**b**) 60%; (**c**) 80%.

Different sizes of grains are observed in samples: the surface of the first PZT element (40% concentration) was rather smooth, with small 5–15 µm diameter accumulations observed on top (Figure 5a). Increasing the PZT concentration to 60% leads to the formation of individual grains (Figure 5b). The surface of 80% PZT piezoelectric film became granular, with smaller grain sizes below 4 µm (Figure 5b). PZT particles loaded in a polyvinyl butyral matrix might be the origin of the irregular shape, nucleation and growth in the solution, thus forming the smaller grain groups. PZT island structures (Figure 5c) were formed where the granular grains surround larger grains. It is also seen that except for a few pinholes, a high deposition density is achieved in 40% and 60% PZT. SEM surface views on a scale of 5–10 μm are presented in Figure 6. Micro cracks of micron length are distributed uniformly in the surface. The element with a PZT concentration of 40% (Figure 6a) has some small structures of about 10–12 nm size. The element with PZT 60% concentration has a small net with cavities formed on the surface (Figure 6b). A higher PZT concentration leads to the formation of three dimensional microstructures with empty cavities of about 6–8 µm diameter (Figure 6c). 

### 3.2. Chemical Composition Novel of Piezoelectric Elements

**Figure 7:**
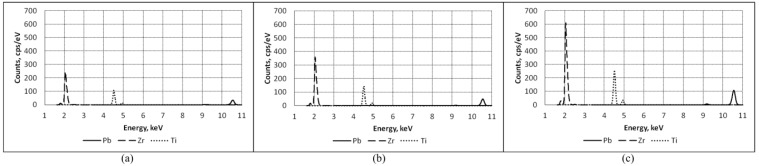
ED spectrum showing peaks of Pb, Zr and Ti of piezoelectric elements with PZT: (**a**) 40%; (**b**) 60% and (**c**) 80%.

The chemical compositions of the cantilever–type piezoelectric elements were investigated by energy-dispersive (ED) spectrometry. A pulse height analysis is employed. Ionization is caused by the incident X-ray photons, and an electrical charge is produced, resulting in the energy dispersive spectrum that is displayed. The X-axis represents the X-ray energy in channels 1.5 to 5 eV wide and the Y-axis represents the number of counts per channel up to 1600 cps/eV. Three main elements were defined in the samples: Lead (Pb), Zirconium (Zr), and Titanium (Ti). Conventionally, for the Ti K_β_ energy resolution peak is specified at about 4.94 keV. For Zr L_α_ the peak is achieved at ~2.05 eV and Pb peak is about 2.35 eV (Figure 7).

For more accurate comparison the peak values of Pb, Zr and Ti of the elements (for 40%, 60% and 80% PZT) are presented in Table 1.

**Table 1:** Peak values of Pb, Zr and Ti of designed piezoelectric elements.

PZT-Concentration	Pb (10.5515 keV)	Zr (2.04236 keV)	Ti (4.50486 keV)
	Intensity Value, cps		
40%	34.49	244.44	107.73
60%	48.62	359.21	143.94
80%	108.05	609.85	256.81

Each element has a defined characteristic peak position. This peak corresponds to the transitions in its electron shell. Chemical analysis shows that the Pb, Zr and Ti concentrations increase proportionally to the concentration of PZT. Both Zr and Ti show dominant K and L peaks, respectively. The Pb spectrum is more complex. Its dominant peak is observed at ~2.35 keV. The intensity value is dependent on the exciting X-ray intensity, on the energy spectrum, the X-ray detector’s efficiency and on the geometry of the investigated element and the source. 

### 3.3. Piezoelectric Properties

**Figure 8:**
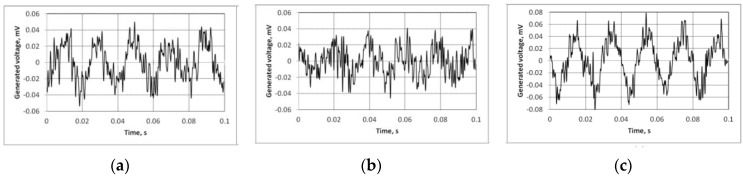
Results of electric potential generated by designed elements with PZT: (**a**) 40%; (**b**) 60% and (**c**) 80%.

A PRISM experimental system was used to investigate the response to electrical excitation of the designed novel cantilever-type piezoelectric elements. At the frequency of 50 Hz and an acceleration of 0.007 g (for open circuit), the element (40% PZT) was able to generate up to 50 μV potential (Figure 8a), and up to 40 μV potential for the element with 60% PZT (Figure 8b). The 80% PZT element generated up to 80 μV electric potential. These results were pre-processed by a 500 Hz low-pass filter.

**Figure 9:**
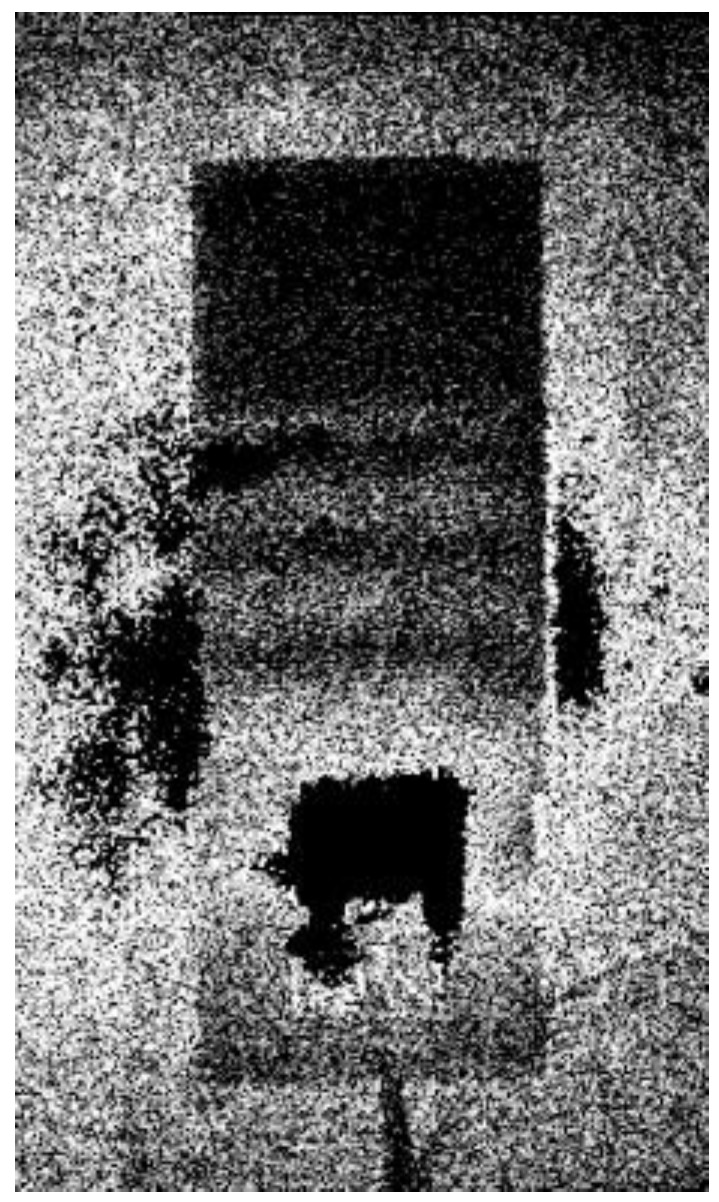
Hologram of the cantilever-type piezoelectric element with PZT 80%.

The cantilever-type piezoelectric element with 80% concentration of PZT therefore shows significant power generation results as a thin layer at low frequencies. The other elements had no signs of any ability to convert electrical potential into mechanical energy. The element with 80% PZT was investigated using the PRISM interferometer of electronic speckle pattern (Figure 9).

The designed element (PZT 80%) was excited by a sinusoidal function with an amplitude of 5 mV at a frequency of 13 Hz (Figure 8). At the first resonant frequency the element vibrates as a clamped-free cantilever resonator in its fundamental flexural mode. A significant advantage of this element is the ability to apply the designed novel piezoelectric composite material at any thickness, form and size on any uniform or non-uniform vibrating surface. 

### 3.4. Periodical Microstructure and SPR

**Figure 10:**
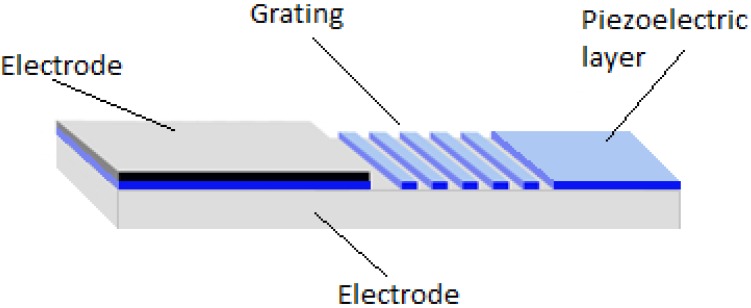
Principal scheme of a cantilever-type piezoelectric sensing platform.

The designed novel cantilever-type piezoelectric element (80% PZT) works in both direct and indirect piezoelectric regimes. The novel piezoelectric composite is a promising material for future experiments. Its unique properties allow a real time and direct observation of affinity interactions, *i.e.*, sensing elements with piezoelectric effect employ the active method for measurements in medical or pharmaceutical fields. Imprinting a periodical microstructure enables to use this platform for studying biomolecular interactions, to analyze functional information, *i.e.*, the information related to physiological effect of an analyte on a living system [15,16,17]. This is essential in many important applications: medicine, pharmacy, cell biology, environmental measurements, etc. For this purpose, a four micron periodical microstructure was imprinted on the formed thin film, made of an 80% concentration of PZT (the element exhibiting the best piezoelectric properties). A schematic view of the designed element is given in Figure 10. The platform consists of a thin piezoelectric film coated on a copper foil working as an electrode. An opposite electrode is formed on the top of the thin film. The periodic microstructure was formed by a hot–embossing procedure under defined conditions.

**Figure 11:**
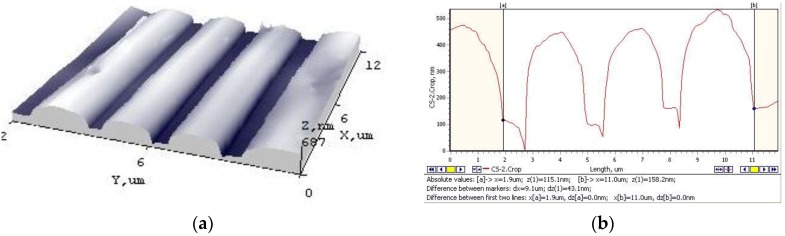
AFM view of a four micron periodical microstructure imprinted on a novel piezoelectric element (**a**) A 3D grating view; (**b**) Topography cross section of the grating.

The surface morphology of the imprinted grating was measured using Atomic Force Microscopy. As previous researches have shown, it is rather hard to imprint a periodic microstructure on a piezoelectric layer because of its brittleness and inelasticity. Here, the additive polyvinyl butyral was chosen to improve these properties. Thus, a well-defined grating was formed (Figure 11). The average grating depth was ~580 nm with an average period of 3.8 µm and rather smooth surface-surface roughness R_q_ = 129.8 nm.

**Figure 12:**
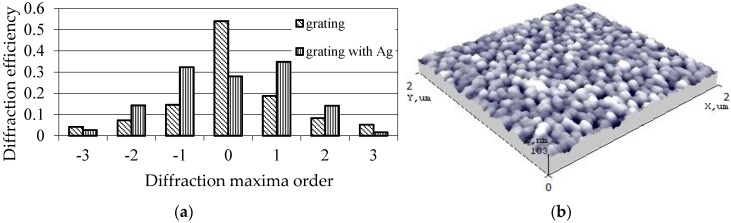
(**a**) Diffraction efficiency measurement results; (**b**) AFM view of a grating top coated with Ag nanoparticles.

The main drawbacks of such a sensing platform arise because of the interaction of biomaterials in the investigated analyte and are strongly influenced by the adsorption of biomolecules, their diameter size and the viscosity of the analyte. To overcome these drawbacks, silver nanoparticles were precipitated on the grating. Previous research [18] has showed that incorporation of Ag greatly increases the optical response of SPR. The SPR enhances the absorption (optical signal); and nanoparticles are used as biological tags for quantitative detection of bio-molecules in analyte. Moreover, a combination of piezoelectric and SPR properties in a single element is an effective way to expand the working range of the element. To prove the relevance of Ag nanoparticles in the designed cantilever-type sensing platform diffraction efficiency measurements were taken (Figure 12a). An AFM surface view of the grating (Figure 12b) showed that approximate diameter of the Ag particles was ~17 nm.

Diffraction efficiency measurements were performed using a laser diffractometer (Figure 12a). A He-Ne red laser light was applied to the grating imprinted on a designed element (80% PZT). Most of the diffraction energy was concentrated on its zero order (~54%) and in its first orders (~33%). For a periodical microstructure with silver nanoparticles, diffracted energy in its zero order has dramatically decreased to ~28% and in the first orders of its maximum increased up to 35%. In second orders of maximum it increased up to 29%. Thus, the results imply that silver nanoparticles significantly improve the optical response of our novel sensing platform.

A future perspective of the designed novel cantilever–type piezoelectric element is related to its integration in MEMS for analysis of functional information such as the physiological effects of an analyte, type and concentration of molecules, *etc.* When, for example, a constant potential is applied, electrochemical reactions may be observed together with the changes of mass or resonant frequency shifts. These measurements are often desired in biomedicine, cell biology, and environmental or pharmacy measurements.

## 4. Conclusions

The designed cantilever-type sensing element made of novel piezoelectric material possesses resonance frequencies and displays higher sensitivity and faster response times. The element works at low frequencies and is able to generate up to 80 μV at 50 Hz frequency. It also works *vice versa*, *i.e.*, at a frequency of 13 Hz with an amplitude of 5 mV and induced by a sinusoidal function it vibrates at the first resonant frequency. The designed element vibrates as a clamped-free cantilever resonator in its fundamental flexural mode.

To obtain a sensing platform suitable for medical applications in MEMS, a four μm periodic microstructure was imprinted. To improve the sensitivity and diffraction efficiency of the designed element silver nanoparticles of ~17 nm diameter were precipitated on the grating surface. This was a result of the appearance of a SPR effect. Incorporation of Ag nanoparticles increases the diffraction efficiency of the grating by 15%–18% in its first orders, and about 10% in its second orders of maxima. The property of piezoelectricity enables one to tune the grating, *i.e.*, to change the grating parameters (grating width and depth) and its optical properties when a voltage is applied. The results show that the designed novel cantilever-type sensing platform exhibits piezoelectric and plasmonic properties. These features are extremely important when designing valuable analytic MEMS instruments for applications such as biomedicine, pharmacy, environment and chemistry. Future experimentations will be related to a practical use of the designed elements in biosensing investigations. 
